# On Coverage of Critical Nodes in UAV-Assisted Emergency Networks

**DOI:** 10.3390/s23031586

**Published:** 2023-02-01

**Authors:** Maham Waheed, Rizwan Ahmad, Waqas Ahmed, Muhammad Mahtab Alam, Maurizio Magarini

**Affiliations:** 1 School of Electrical Engineering and Computer Science, National University of Sciences and Technology (NUST), Islamabad 44000, Pakistan; 2 Pakistan Institute of Engineering and Applied Sciences (PIEAS), Islamabad 45650, Pakistan; 3 Thomas Johann Seebeck Department of Electronics, Tallinn University of Technology, 19086 Tallinn, Estonia; 4 Dipartimento di Elettronica, Informazione e Bioingegneria, Politecnico di Milano, 20133 Milano, Italy

**Keywords:** unmanned aerial vehicle, emergency network, critical nodes, coverage, capacity, age of information, reinforcement learning

## Abstract

Unmanned aerial vehicle (UAV)-assisted networks ensure agile and flexible solutions based on the inherent attributes of mobility and altitude adaptation. These features render them suitable for emergency search and rescue operations. Emergency networks (ENs) differ from conventional networks. They often encounter nodes with vital information, i.e., critical nodes (CNs). The efficacy of search and rescue operations highly depends on the eminent coverage of critical nodes to retrieve crucial data. In a UAV-assisted EN, the information delivery from these critical nodes can be ensured through quality-of-service (QoS) guarantees, such as capacity and age of information (AoI). In this work, optimized UAV placement for critical nodes in emergency networks is studied. Two different optimization problems, namely capacity maximization and age of information minimization, are formulated based on the nature of node criticality. Capacity maximization provides general QoS enhancement for critical nodes, whereas AoI is focused on nodes carrying critical information. Simulations carried out in this paper aim to find the optimal placement for each problem based on a two-step approach. At first, the disaster region is partitioned based on CNs’ aggregation. Reinforcement learning (RL) is then applied to observe optimal placement. Finally, network coverage over optimal UAV(s) placement is studied for two scenarios, i.e., network-centric and user-centric. In addition to providing coverage to critical nodes, the proposed scheme also ensures maximum coverage for all on-scene available devices (OSAs).

## 1. Introduction

Agility, fast deployment, and unprecedented coverage are potential prospects for emergency communication networks. The terrestrial networks are often vulnerable to unexpected and emergency events, such as earthquakes, floods, or terrorist attacks. Moreover, they lack mobility and altitude adaptation to provide eminent coverage. This completely hinders or severely disrupts the information dissemination to public safety responders and security agencies to perform the desired relief and rescue work.

UAV-assisted communication is considered a promising solution for emergency networks. UAV-assisted emergency networks (ENs) have the potential to accelerate rescue efforts owing to their inherent characteristics of fast deployment, flexible infrastructure, mobility, and coverage areas [1]. UAVs can efficiently detect and recognize situations from high altitudes or long distances [2]. According to the Research and Markets Report, the public safety drone market is expected to reach USD 1.15 billion worldwide by the end of 2022 [3]. Coherent Market Insights forecasts the global safety and security drones’ market to grow at a CAGR of 34.7% over 2019–2027, which was estimated to be USD 354.8 million in 2019 [4]. Meanwhile, the authors in [5] recommend that more research is required in order to implement UAV-assisted remote sensing in disaster regions.

### 1.1. UAV-Assisted Communication

Recently, UAV-assisted communication has garnered a lot of interest. They can be deployed as independent pieces of equipment for ubiquitous coverage or as part of larger networks for relaying, offloading, information dissemination, data collection, and surveillance. UAV-assisted communications mostly span relaying [6], offloading [7], load balancing [8], energy harvesting [9], etc. Unlike conventional networks, ensuring communication in UAV-assisted networks is constrained by multiple factors [10], such as line-of-sight/non-line-of-sight (LoS/NLoS) links, node distribution and behavior, and data and power transmission strategies [11]. LoS/NLoS propagation-based UAV-assisted cellular networks are studied in [12]. Moreover, the efficacy of such a deployment is dependent on the number of UAVs and their placement. The optimized placement of UAV relays for dual and multi-hop UAV links is studied in [13]. Mobility-aware UAVs’ performance in mobile edge computing is studied/optimized using deep reinforcement learning in [14]. UAVs are specifically deployed where terrestrial networks do not meet the coverage and rate constraints. Optimal UAV placement ensures enhanced coverage, improved capacity, and effective QoS of the network. Optimal UAV altitude to ensure maximum coverage is studied in [15,16]. Capacity analysis over random UAV trajectories is performed in [17]. Capacity enhancement based on UAV placement is studied for relaying networks in [18], wireless sensor networks (WSNs) in [19], and OFDM network in [20]. Most of the existing literature on UAV placement often assumes UAVs to be a part of a well-defined network where their placement and coverage regions are readily defined/optimized.

### 1.2. UAV-Assisted Emergency Networks

UAV-assisted emergency networks, however, demand extra communication design parameters as the underlying network differs from the conventional well-defined network. UAVs in emergency scenarios do not have prior information about placement, coverage, user density, user classification, etc. Therefore, the aforementioned techniques provide limited insight into the placement of UAVs in emergency networks. Coverage maximization based on the altitude of UAVs in an emergency network is studied in [21]. The capacity and overall outage probability for fixed altitude UAVs acting as relays for on-scene available devices (OSAs) in emergency scenarios are analyzed in [22]. Optimized UAV placement is discussed for periodically gathering information over multi-zone disaster regions in [23]. A similar study optimizing UAV placement to maximize the number of covered OSAs with different quality-of-service (QoS) requirements is carried out in [24]. Further, computational intelligence techniques for optimized placement to ensure coverage and QoS in an emergency network are employed in [25].

The basic aim of a UAV-assisted EN is to enable reliable and timely information flow from on-scene available devices OSAs to command control [26]. The freshness of data is a prominent feature in ensuring effective relief and rescue operations. Age of information (AoI) serves as an important factor in such situations, ensuring the freshness of data. AoI minimization in WSNs and Internet of things (IoTs) is studied in [27,28]. UAV trajectory design to ensure the timeliness of information in an emergency network is evaluated in [29]. However, not much literature is available on AoI minimization concerning emergency networks. Although some of the existing literature focuses on UAV placement based on capacity and AoI of UAV-assisted emergency networks, the presence of critical nodes (CNs) is overlooked. Most of the literature on emergency networks considers an OSA to be a conventional network device that can be classified based on its QoS requirement. However, this approach is inconsequential in the context of emergency networks.

### 1.3. Critical Nodes in Emergency Networks

Critical nodes can be identified as the ones carrying crucial information that needs to be shared with command control in a reliable and timely manner. As the emergency networks differ from trivial setups, it is often observed that certain OSAs, termed critical nodes, carry indispensable information i.e., more decisive in comparison to other OSAs’ information. For instance, in case of a terrorist attack, an OSA in the vicinity of a terrorist can relay more meaningful information than other OSAs. Such a scenario cannot be handled by treating that particular node as a conventional OSA. In addition, the unavailability/lack of on-spot device charging facilities may hinder data acquisition. The OSAs carrying crucial information with low battery levels require urgent connectivity to avoid any setback in rescue operations. The efficacy of rescue and relief operations highly depends on the timeliness and accuracy of knowledge about the affected region. Therefore, the placement of UAV(s) based on a conventional set of OSAs is not a rational approach in emergency networks. A disaster situation in [30] incorporates 2D UAV placement in the presence of critical nodes, whereas the efficiency in UAV networks highly depends on LoS/NLoS probabilities and elevation-demanding 3D optimization. A secondary critical challenge in certain emergency networks is to secure this communication link as discussed in [31,32].

### 1.4. Contribution of Work

**Figure 1:**
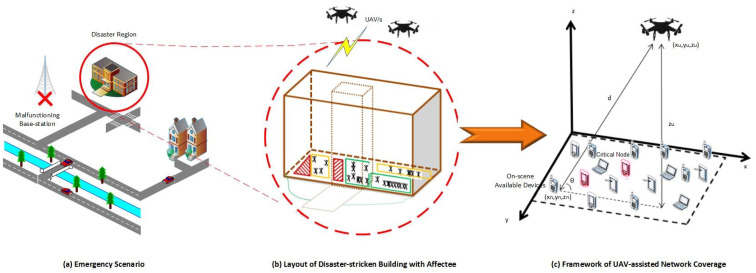
System model.

In this work, the 3D optimized placement for a multi-UAV emergency network is studied over a disaster region for critical nodes. The emergency situation and network are depicted in Figure 1. As the terrestrial network is destroyed, the area is deprived of network coverage. The UAV-assisted EN is deployed to connect the OSAs to the outside world. Several CNs are detected among these OSAs, which may have critical information, critical battery levels, or both. The presence of CNs and OSAs in the network is analyzed from two aspects: (i) capacity and (ii) age of information [33].

A capacity maximization problem is formulated to improve the network capacity for CNs, whereas an AoI minimization problem is formulated to ensure the freshness and timeliness of data retrieval from CNs. For comparison, two schemes are considered: random placement and equal region partitioning (ERP). The results for both problems show prominent improvement compared to baseline schemes for an increased number of UAVs. Further, coverage to OSAs is analyzed for optimally placed UAVs. In addition to providing coverage to critical nodes, the proposed scheme ensures maximum coverage of other OSAs as well. Specifically, in this paper, the following contributions are made:

- Initially, capacity maximization and AoI minimization problems are formulated from CNs’ perspective in an emergency network. A reinforcement learning (RL) framework, along with various partitioning techniques, is employed to optimize the UAV(s) placement for the capacity and AoI optimization problems.
- The results of different partitioning schemes are then compared and analyzed in terms of capacity, AoI, and computational complexity.
- Furthermore, the coverage region of optimally placed UAV(s) is analyzed, specifically for CNs and generally for overall OSAs, over two scenarios, i.e., network-centric and user-centric.

The rest of the paper is arranged in the following manner. The system model and channel specifications are described in Section 2. The problem formulation, region partitioning, and RL framework are presented in Section 3. The simulation results for capacity maximization and AoI minimization problems are shown in Section 4 for various partitioning techniques. It also covers the performance for network-centric and user-centric scenarios. Finally, the conclusion is drawn at the end.

## 2. System Model

Consider a situation in which *N* OSAs, represented as a set N, are randomly distributed in a disaster region. These OSAs are indexed by n∈N with coordinates $\left(x_{n},y_{n},z_{n}\right)$. Among N OSAs, certain nodes are critical and represented as a set $N_{\mathrm{CN}}$, such that $N_{\mathrm{CN}}\subseteq N$. The set $N_{\mathrm{CN}}$ is indexed by $n_{CN}$. The network coverage is provided by *U* UAVs with coordinates $\left(x_{u}^{\prime },y_{u}^{\prime },z_{u}^{\prime }\right)$, where u∈{1,2,…,U}. Both the uplink and downlink consist of $n_{sub}$ subcarriers. The bandwidth of each subcarrier is defined as *W*. The $n_{sub}$ subcarriers are exclusively assigned to a set of $N_{u}$ (indexed by $n_{u}$) OSAs associated with $u^{\mathrm{th}}$ UAV in its coverage area. It is assumed that all channels exhibit both path loss and fading [13,15]. The path loss between $n^{\mathrm{th}}$ OSA and $u^{\mathrm{th}}$ UAV is defined as:

(1) $$PL_{n,u}\left(d\right)=\alpha 10\log_{10}d+\beta$$

where $d=\sqrt{{\left(x_{u}^{\prime }-x_{n}\right)}^{2}+{\left(y_{u}^{\prime }-y_{n}\right)}^{2}+{\left(z_{u}^{\prime }-z_{n}\right)}^{2}}$, α is the pathloss exponent, and β is the path loss at the reference point. The value of pathloss greatly depends on the altitude $z_{u}^{\prime }$, elevation angle θ, and certain environmental factors, such as *a* and *b*.

(2) $$\beta =B+\frac{A}{1+aexp(-b[\theta -a])}$$

where *A* and *B* are the additional attenuation factors for LoS and NLoS connections given by $A=\eta_{LoS}-\eta_{NLoS}$ and $B=10\log_{10}{\left(\frac{4\pi f}{c}\right)}^{2}+\eta_{LoS}$, $\eta_{LoS}$ and $\eta_{NLoS}$ depend on propagation environment, *f* is the carrier frequency, *c* specifies the speed of light, and $\theta =\frac{180}{\pi }\arcsin\left(\frac{z_{u}^{\prime }}{d}\right)$.

The signal to interference plus noise ratio (SINR) $\gamma_{n_{u},u}$ for $n_{u}$ thus becomes

(3) $$\gamma_{n_{u},u}=\frac{\sum_{k=1}^{n_{sub}}\sigma_{n_{u},u}\left(k\right)p_{u}{\left|g_{n_{u},u}\left(k\right)\right|}^{2}PL_{n_{u},u}^{-1}}{\sum_{k=1}^{n_{sub}}\sigma_{n_{u},u}\left(k\right)\left(WN_{0}+I_{n_{u},u}\left(k\right)\right)}\forall n_{u}\in N_{u},$$

where $\sigma_{n_{u},u}\left(k\right)$ is an indicator function of subcarrier assignment to the OSA $n_{u}$ of the $u^{\mathrm{th}}$ UAV. If a subcarrier is assigned to $n_{u}$ then $\sigma_{n_{u},u}\left(k\right)=1$, and 0 otherwise. $p_{u}$ is the transmit power of $u^{\mathrm{th}}$ UAV, $|g_{n_{u},u}|$ follows Nakagami distribution with variance Ω, $N_{0}$ is the thermal noise power, and $I_{n_{u},u}$ is the interference given by (4).

(4) Inu,u(k)=∑u′=1u≠u′U∑nu′∈Nu′∑k=1nsubσnu′,u′(k)ρnu,u′(k)

where $\rho_{n_{u},u^{\prime }}\left(k\right)=\frac{p_{u^{\prime }}{\left|g_{n_{u},u^{\prime }}\left(k\right)\right|}^{2}}{PL_{n_{u},u^{\prime }}}$

In this paper, the OSA’s association with UAV(s) is based on the minimum pathloss criterion. The indicator function can only be 1 if the minimum pathloss is less than a given threshold.

The overall network capacity *C* is defined by (5).

(5) $$C=\sum_{u=1}^{U}\sum_{n_{u}=1}^{N_{u}}\sum_{k=1}^{n_{sub}}\sigma_{n_{u},u}\left(k\right)W\log_{2}\left(1+\gamma_{n_{u},u}\right)$$

## 3. Problem Formulation and Optimized 3D Placement

The point of interest in this work is the placement of UAV(s) to maximize the capacity of critical nodes $C_{CN}$; therefore, an optimization problem is defined by (7). In a similar manner to (5), the capacity of CNs is given as:

(6) $$C_{CN}=\sum_{u=1}^{U}\sum_{n_{CN}\in N_{CN}}\sum_{k=1}^{n_{sub}}\sigma_{n_{CN},u}\left(k\right)W\log_{2}\left(1+\gamma_{n_{CN},u}\right)$$

The capacity maximization problem of CNs constrained by the location of UAV(s) in the network is defined by

(7a)maxxu′,yu′,zu′CCN(7b)s.t.xu,min′ ≤ xu′ ≤ xu,max′,(7c)yu,min′ ≤ yu′ ≤ yu,max′,(7d)zu,min′ ≤ zu′ ≤ zu,max′,(7e)CnCN,u ≥ RnCN ∀ nCN,(7f)PLnu,u ≤ PLth ∀ nu,

where (7b)–(7d) represent the 3D placement constraints, (7e) ensures coverage to all the CNs based on rate requirement $R_{n_{CN}}$ and (7f) indicates the pathloss threshold value $PL_{th}$ for overall network nodes. Minimum pathloss criterion-based coverage would be added specifically with this constraint. After $PL_{th}$, the node $n_{u}$ no longer remains connected to UAV *u*. The methodology for the capacity maximization problem is described in Algorithm 1.
**Algorithm 1** Framework for Capacity Maximization**Require:** System Parameters $\left(PL_{n_{CN},u},W,\sigma_{n_{CN},u},p_{u},p_{n_{CN},u},N_{0}\right)\forall n_{CN}\in N_{\mathrm{CN}}$  Find Optimal UAV placement ($x_{u}^{\prime },y_{u}^{\prime },z_{u}^{\prime }$) by solving Equation (7) **return** Maximum Capacity for CNs.


To formulate the AoI minimization problem, the average AoI of the data collected from critical nodes ${\bar{\Delta }}_{CN}$ is given by (8)

(8) $${\bar{\Delta }}_{CN}=\frac{1}{N_{CN}}\sum_{u=1}^{U}\sum_{n_{CN}\in N_{CN}}\sum_{k=1}^{n_{sub}}\sigma_{n_{CN},u}\left(k\right)t_{n_{CN},u}\left(k\right)$$

where $t_{n_{CN},u}$ is the data-uploading time by $n_{CN}$ to *u* [33]. The uploaded data during hovering of UAV is the expectation taken over distance-dependent pathloss, which is thus impacted by UAV placement. The indicator function $\sigma_{n_{CN},u}\left(k\right)$ defines the subcarrier assignment of $n_{CN}$ connected to $u^{\mathrm{th}}$ UAV.

The AoI minimization problem is defined as (9).

(9a)minxu′,yu′,zu′ΔCN—(9b)s.t.tnCN,uCnCN,u ≥ DnCN,u,(9c)(7b) − (7d),

where (9b) constraints the amount of data $D_{n_{CN},u}$ uploaded by CN $n_{CN}$ to UAV *u* in time $t_{n_{CN},u}$. The methodology for the AoI minimization problem is described in Algorithm 2.
**Algorithm 2** Framework for AoI Minimization**Require:** System Parameters ($PL_{n_{CN},u},W,\sigma_{n_{CN},u},p_{u},p_{n_{CN},u},D_{n_{CN},u},N_{0})\forall n_{CN}\in N_{\mathrm{CN}}$   Find Optimal UAV placement ($x_{u}^{\prime },y_{u}^{\prime },z_{u}^{\prime }$) by solving Equation (9) **return** Minimum average AoI for CNs.


Apart from capacity enhancement and AoI minimization for critical nodes, the coverage to all CNs is also ensured. The steps to analyze network coverage are elaborated in Algorithm 3. Two parameters are defined to analyze network coverage, i.e., PL coverage and rate coverage. The overall coverage region is the overlap of these two. The PL coverage probability $P_{cvg,PL}$, at a given pathloss threshold τ, is given by:

(10) $$P_{cvg,PL}\left(\tau \right)=P\left(PL_{n}<\tau \right)\forall n_{u},n_{CN}$$

Similarly, the rate coverage probability $P_{cvg,R}$ is calculated, for a given rate requirement or threshold Γ, as:

(11) $$P_{cvg,R}\left(\Gamma \right)=P\left(C_{n}>\Gamma \right)\forall n_{u},n_{CN}$$

The objective functions of (7) and (9) can not be solved directly over $x_{u}^{\prime }$, $y_{u}^{\prime }$, and $z_{u}^{\prime }$. Therefore, a two-step approach is adopted to get the optimal solution. At first, the regions for multi-UAV placement are identified. Fine adjustment in the position of the UAV(s) is carried out using RL.
**Algorithm 3** Framework for Network Coverage**Require:** ($x_{u}^{\prime },y_{u}^{\prime },z_{u}^{\prime }$), $p_{u},p_{n,u},\tau ,\Gamma ,R_{n}$  Find Coverage based on (10) and (11)  **return** Coverage of CNs and OSAs over optimized placement.


### 3.1. Region Partitioning

In this step, for all the objective functions, the disaster region is partitioned into non-overlapping and distinct sub-regions. The number of sub-regions is defined by the number of UAVs so that each UAV serves one sub-region containing a cluster of OSAs. For instance, for U=4, four sub-regions are formed with each sub-region covered by a UAV *u*. Two methods for region partitioning are employed, namely K-mean clustering partition (KP) and a customized partition (CP).

In KP, the clusters or sub-regions are formed by minimizing the sum of the squared distance between the OSAs and the cluster’s centroid [34]. The KP approach does not rely on location information of UAVs. In effect, it only uses 2D information of OSAs. However, in CP, the partitioning requires the location information of UAV and the OSAs. Unlike KP, which only uses distance information between OSAs, the partitioning in CP is carried out while considering both LoS/NLoS probabilities and the elevation of UAVs. An exhaustive search is performed over the region in three dimensions to find CP. In CP, the sum minimum pathloss criterion is used. It is possible that some of the OSAs are not covered by any UAV considering (7f).

For both types of partition, the UAV(s) placement over the partitioned region is found using both the K-mean centroid and RL methods. This leads to four cases:

- UAV(s) placement by calculating centroid in KP: The UAV(s) $x_{u}^{\prime }$ and $y_{u}^{\prime }$ coordinates are calculated based on cluster’s centroid, whereas the altitude is varied between $z_{u,min}^{\prime }\leq z_{u}^{\prime }\leq z_{u,max}^{\prime }$ to satisfy the objective functions (7) and (9).
- UAV(s) placement with RL over KP: A 3D RL technique is applied over the KP partitioned sub-regions to obtain the placement of UAV(s), satisfying the respective objective functions.
- UAV(s) placement by calculating centroid in CP: The UAVs $x_{u}^{\prime }$ and $y_{u}^{\prime }$ are calculated based on cluster’s centroid in CP partitioned sub-regions, whereas the altitude is varied between $z_{u,min}^{\prime }\leq z_{u}^{\prime }\leq z_{u,max}^{\prime }$ to satisfy the objective functions (7) and (9).
- UAV(s) placement with RL over CP: A 3D RL technique is applied over the already-partitioned CP sub-regions to obtain the placement of UAV, satisfying the objective functions.

#### Equal Region Partitioning (ERP)

Another partition termed equal region partitioning (ERP) is considered for comparison purposes. In ERP, the disaster region is divided into *U* regions of equal area. The UAVs are placed at the center of these equally partitioned regions and their altitude is derived from RL over CP placement method. For instance, if a single UAV is to be placed in a 100 m × 100 m region, then $\left(x_{u}^{\prime },y_{u}^{\prime }\right)=\left(50,50\right)$.

### 3.2. RL Framework

The placement of each UAV is obtained using a low complexity Q-Learning technique where the UAV acts as an agent. At each iteration, the agent is in a state s∈S performing an action a∈A according to the policy π to maximize the reward *r*. At any point, the state of UAV $u_{s}$ is specified by $\left(x_{u}^{\prime },y_{u}^{\prime },z_{u}^{\prime }\right)$, and the action space is defined as A={forward,backward,up,down,ascend,descend}. A reward is calculated when the UAV transitions from one state to the next state $s^{\prime }$ after performing a random action. The new location of the UAV $u_{s^{\prime }}\leftarrow \left(u_{s},a\right)$ depends on its previous state and action taken according to conditional transition probability $P(s^{\prime },r|s,a)$. The reward is chosen under some prominent network performance metrics. In the current case, it is defined by capacity and AoI for their respective optimization problems. The reward structure for the capacity maximization problem is defined and calculated by (12).

(12) $$r_{C}=\left\{\begin{array}{cc} 1 & \mathrm{if}C_{new}\geq C_{previous}\\ 0 & \mathrm{if}C_{new}=C_{previous}\\ -1 & \mathrm{if}C_{new}\leq C_{previous} \end{array}\right.$$

A similar function is defined for AoI in (13):

(13) $$r_{\bar{\Delta }}=\left\{\begin{array}{cc} 1 & \mathrm{if}{\bar{\Delta }}_{new}\leq {\bar{\Delta }}_{previous}\\ 0 & \mathrm{if}{\bar{\Delta }}_{new}={\bar{\Delta }}_{previous}\\ -1 & \mathrm{if}{\bar{\Delta }}_{new}\geq {\bar{\Delta }}_{previous} \end{array}\right.$$

The objective is to maximize the reward in a given interval while aiming to maximize the long-term reward over all future intervals:

(14) $$Q^{\pi }\left(s,a\right)=E\left[\sum_{i=I}^{\infty }\psi^{i-I}r\left(I\right)\right]$$

where E[.] is the expectation over discounted reward, ψ is discount factor, and r(I) is instantaneous reward.

The optimization problems can be reformulated as (15).

(15a)maxs,aQπ(s, a)(15b)s.t.s∈S, a∈A

All subsequent actions are selected based on the greedy policy [35]. At each iteration, the state–action pair is updated according to (16) with learning rate δ and discount factor ψ, generating a tuple <s,a,r,Q>.

(16) $$Q_{new}\left(s,a\right)\leftarrow Q\left(s,a\right)+\delta \left[r\left(s,a\right)+\psi max_{a}Q\left(s^{\prime },a\right)\right]$$

The RL approach converges when the change in $Q_{new}\left(s,a\right)$ during the subsequent iterations becomes negligible. Algorithm 4 depicts the working of the proposed RL framework.
**Algorithm 4** Q-Learning for UAV Placement**Require:** Learning Rate δ, Discount Factor ψ, Reward Structure $r_{C},r_{\bar{\Delta }}$     Region Partition based on KP and CP Initialize Q(s, a) ∀s∈S, a∈A arbitrarily      Select random *s* **repeat** (for each step of episode):     choose *a* from *s* using policy derived from *Q* to obtain maxQ;     take action *a*, observe *r*, update *s*;     $Q_{new}\left(s,a\right)\leftarrow Q\left(s,a\right)+\delta \left[r\left(s,a\right)+\psi max_{a}Q\left(s^{\prime },a\right)\right]$;     $s\leftarrow s^{\prime }$; **until**
*s* is terminal    (no more learning episodes) **return** evaluation results.


### 3.3. Computational Complexity

The simplest and most reliable algorithm to find optimal values is considered to be the exhaustive search. It examines every possible element inside the search region providing the best possible match, thus proving to be stable and optimal. However, for the present scenario, its complexity would be $O(IUN^{U})$ over I iterations for *U* number of UAVs and *N* OSAs, demanding a more effective algorithm.

In region partitioning, the KP and CP algorithms are applied. Both algorithms have the computational complexity of O(IUN). The performance of KP is dependent on the initial center, is deterministic over the runs and is regarded as non-stable and suboptimal. Further, the optimal placement is determined using Q-learning through an iterative process. Its computational complexity is similar to K-mean, i.e., O(IUN) when the Q-table is updated through the training process. It is a stable but suboptimal algorithm.

The ERP scheme used for comparison purposes involves basic arithmetic operations; thus, it is considered a constant–time operation with complexity O(1). However, the scheme would only be effective for symmetrical regions and would not work with amorphous boundaries.

## 4. Simulation and Performance Evaluation

A disaster region of 100 m × 100 m, with 100 randomly distributed OSAs, is considered. Among these, 15 are randomly chosen to act as CNs. UAV(s) are deployed to retrieve information for first responders. The transmit power for a UAV(s) is set at 0.5 W and the nodes could transmit at 0.125 W. The bandwidth provided to each tier is 20 MHz and each band is divided into 64 subcarriers. Round-robin scheduling is applied to assign subcarriers to the nodes. The results are obtained by employing extensive Monte Carlo simulations in MATLAB and the simulation parameters are given in Table 1 [36].

At first, an optimal location for a single static UAV is obtained based on capacity maximization and AoI minimization subject to the distribution of CNs. The placement decision is made either by the UAV, if it has enough computational capacity, or by the command control. The disaster region is then divided into *U* sub-regions to optimize the placement of multiple UAVs. Each sub-region is governed by a single UAV. The results are studied over the aforementioned region partitions. The observations are carried out spanning a range of coordinates, i.e., $\left\{x_{u,min}^{\prime },\ldots ,x_{u,max}^{\prime }\right\}$, $\left\{y_{u,min}^{\prime },\ldots ,y_{u,max}^{\prime }\right\}$, and $\left\{z_{u,min}^{\prime },\ldots ,z_{u,max}^{\prime }\right\}$ to find optimal location coordinates of UAV(s) $\left(x_{u}^{\prime },y_{u}^{\prime },z_{u}^{\prime }\right)$.

**Figure 2:**
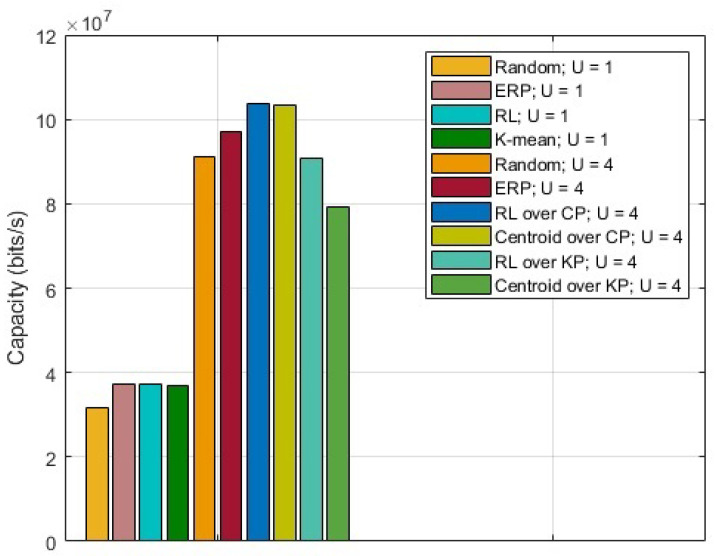
Capacity for CNs.

The results for the capacity maximization problem are shown in Figure 2. For comparison, random placement and ERP are also presented. ERP acts as a middle ground between random placement and proposed RL over CP. It is observed that the UAV(s) placement with RL over CP provides the highest capacity. Based on region partition, for U=4, it is observed that the capacity obtained by calculating centroids in KP is 56.06% lower than the one obtained by calculating centroids in CP, whereas introducing RL reduces that difference to 13.86%.

**Figure 3:**
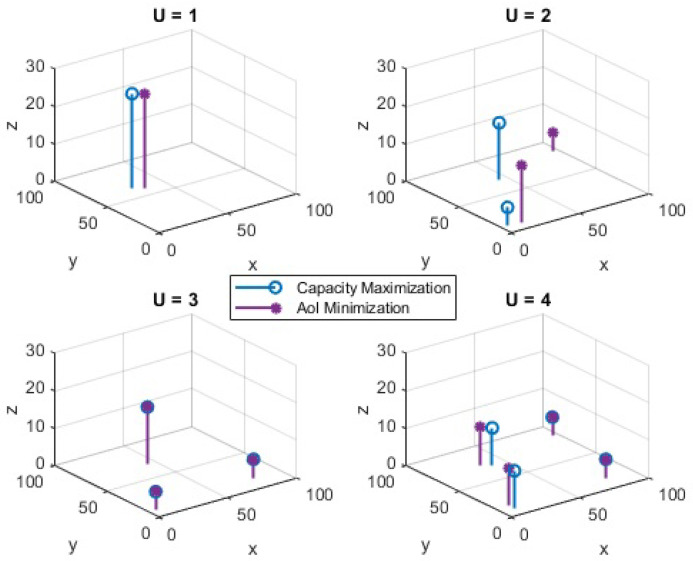
Placement optimization.

**Figure 4:**
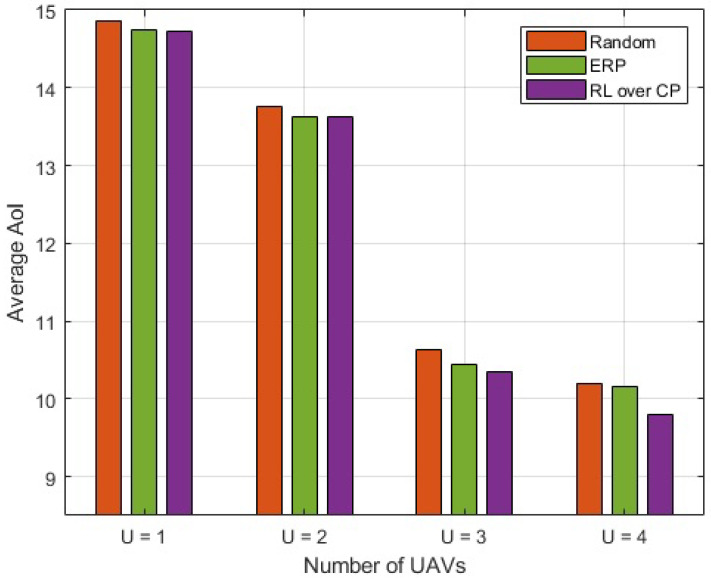
Average AoI of CNs.

The optimized UAV placement from individual optimization problems, namely capacity maximization and AoI minimization, are shown in Figure 3. Considering the capacity performance in Figure 2, the results are only shown for UAV(s) placement with RL over CP. The number of UAVs varies from 1 to 4. It can be seen that when the number of UAVs is lower than 3, the UAV positions for capacity and AoI are different. However, when the number of UAVs is increased, the UAVs converge to an almost similar position for capacity and AoI. In general, when the number of UAVs is large, the UAV placement for the above problems is found to be significantly close to each other and, in some situations, it overlaps. The average optimized AoI for different numbers of UAVs is depicted in Figure 4. It is observed that with an increase in the number of UAVs, the difference between the AoI of RL over CP, and ERP increases. The difference becomes significant when the number of UAVs is increased beyond 2. With U=4, the average AoI of RL over CP is 3.692% reduced compared to ERP. It is to be noted that for fewer UAVs, ERP shows an improved performance compared to random placement but almost similar to RL over CP; however, as the number of UAVs increases, it becomes closer to random placement. Therefore, for fewer UAVs it is favorable to deploy ERP and avoid complexity; however, as the number of UAVs increases, RL over CP is the best option.

**Figure 5:**
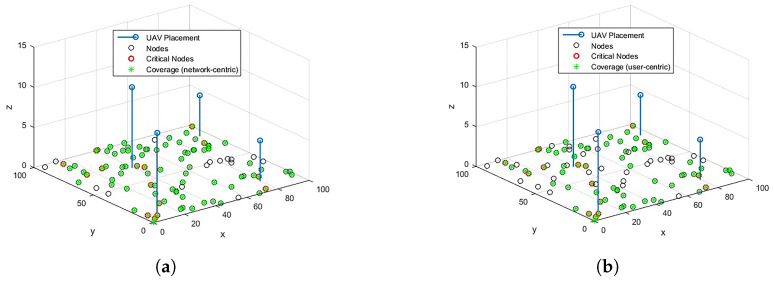
Coverage over optimized placement. (**a**) Coverage: network-centric. (**b**) Coverage: user-centric.

**Figure 6:**
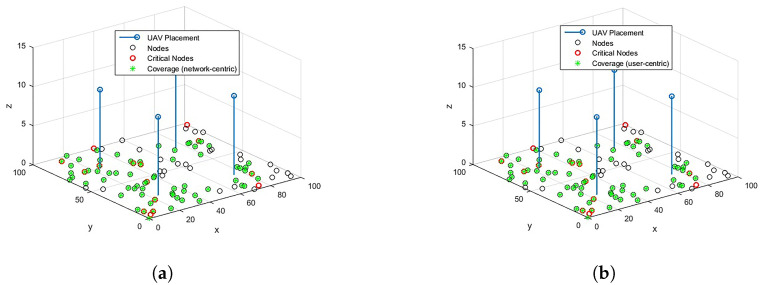
Coverage over ERP. (**a**) Coverage: network-centric. (**b**) Coverage: user-centric.

**Figure 7:**
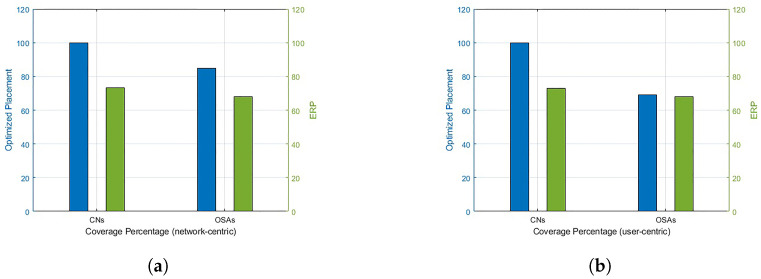
Coverage: optimized placement vs. ERP. (**a**) Coverage: network-centric. (**b**) Coverage: user-centric.

For RL over CP-based 3D optimal locations for U=4, the coverage results for network-centric and user-centric scenarios are depicted in Figure 5. The results for ERP are also shown in Figure 6 for comparison. The network-centric scenario assumes that all the OSAs have the same data rate requirement, $R_{n}=1$ Mbps, whereas in a user-centric scenari, o the data rates $R_{n}$ are user-specific. The data rates are randomly assigned to the OSAs from set $R_{n}$. With U=4, 100% coverage to CNs and 85% coverage to overall network OSAs can be provided through optimized placement. In the user-centric case, although the coverage of overall network OSAs is reduced to 68%, the coverage to all the CNs is still guaranteed. A comparison of the proposed scheme with baseline ERP is provided in Figure 7. It is noted that RL over CP-based placement provides 26.7% improved coverage to CNs in both cases compared to ERP. The coverage to the overall network is also reduced by 16% in the network-centric case, whereas the reduction for user-centric cases is negligible.

## 5. Conclusions

This research aims to maximize the capacity and minimize the average AoI for CNs using optimized UAV placement in UAV-assisted EN. The problem is simplified through the partitioning of the disaster region using KP and CP approaches. Subsequently, the required goal is achieved using RL to derive the 3D optimal placement of UAV(s) over partitioned regions. The results indicate that RL over CP provides 6.68% improved capacity and 3.692% reduced average AoI compared to ERP. The result of the proposed scheme shows visible improvements compared to random placement for all scenarios. In comparison to ERP, the improvement is negligible for a lower number of UAVs. However, as the number of UAVs increases, prominent improvement is observed for the proposed RL over CP scheme. It is also observed that as the number of UAVs increases, the optimal locations for both capacity maximization and AoI minimization problems converge. The AoI minimization requires that a significant number of UAVs must be deployed. However, this may lead to an increased interference in EN. Finally, the optimal placement provides unprecedented coverage to critical nodes, along with 85% coverage to overall network nodes. It is also observed that the performance of ERP is ostensibly reduced compared to RL over CP. It covers 26.6% fewer CNs and 16% fewer overall OSAs compared to the proposed scheme. The work provides the performance indicators for the basic platform to start with complex optimization problems. The work in its current form is limited to simulations and not extended to experiments. Future tasks, with regards to simulation, include but are not limited to joint capacity and AoI optimization, implementation of mobile UAVs, training for location information, and backhaul limitation parameters. Further, future work involves conducting real experiments based on this study and its extended simulation versions. In addition, securing communication links for certain emergency networks will be explored further.

## Figures and Tables

**Table 1:** Simulation parameters.

Coverage Area	100 m × 100 m
*N*	100
*f*	2 GHz
*W*	20 MHz
$n_{sub}$	64
α	2
$\left(\eta_{LoS},\eta_{NLoS}\right)$	(0.1,21) dB
a,b	5.0188,0.3511 (suburban)
$p_{n,u}$	0.125 W
$p_{u}$	0.5 W
$N_{0}$	−174 dBm/Hz
$\left(x_{u,min}^{\prime },y_{u,min}^{\prime },z_{u,min}^{\prime }\right)$	(0,0,0)
$\left(x_{u,max}^{\prime },y_{u,max}^{\prime },z_{u,max}^{\prime }\right)$	(100,100,30)
$R_{n}$	[0.5,1.0,1.5,2.0] Mb/s

## Data Availability

Not applicable.

## Abbreviations

The following abbreviations are used in this manuscript:

UAV	Unmanned aerial vehicle
ENs	Emergency networks
CNs	Critical nodes
QoS	Quality-of-service
WSNs	Wireless Sensor Networks
OFDM	Orthogonal Frequency-Division Multiplexing
IoTs	Internet of Things
AoI	Age of information
RL	Reinforcement learning
LoS	Line-of-sight
NLoS	Non-line-of-sight
OSAs	On-scene available devices
SINR	Signal to interference plus noise ratio
KP	K-mean clustering
CP	Customized partition
ERP	Equal region partitioning
